# Supplementary material for: Modelling a Western Lifestyle in Mice: A Novel Approach to Eradicating Aerobic Spore-Forming Bacteria from the Colonic Microbiome and Assessing Long-Term Clinical Outcomes
Source: Biomedicines. 2024 Oct 7;12(10):2274. doi: 10.3390/biomedicines12102274 (PMC11504893; doi:10.3390/biomedicines12102274)
Supplement: Supplementary file 1 [file biomedicines-12-02274-s001.zip › biomedicines-3183481-supplementary.pdf]

Table S1. Sequence of primers used for RT-Qpcr. **House Keeping Gene*

Gene	Primer type	Sequence (5′ - 3′)
Beta-Actin*		
	Sense	CTGTCCCTGTATGCCTCTG
	Anti-sense	ATGTCACGCACGATTTCC
IL-10		
	Sense	AAGGCAGTGGAGCAGGTGAA
	Anti-sense	CCAGCAGACTCAATACACAC
TLR-2		
	Sense	CTGAAGCTGTTGCGTTACAT
	Anti-sense	ACTACGTCTGACTCCGAGGG
TLR-4		
	Sense	TTCAGAGCCGTTGGTGTATC
	Anti-sense	CCCATTCCAGGTAGGTGTTT
TNF-α		
	Sense	CACCACGCTCTTCTGTCTACTG
	Anti-sense	TGAGAAGATGATCTGAGTGTGA
IL-1β		
	Sense	CAGGATGAGGACATGAGCACC
	Anti-sense	CTCTGCAGACTCAAACTCCAC
IL-6		
	Sense	AGTTGCCTTCTTGGGACTGA
	Anti-sense	TCCACGATTTCCCAGAGAAC
Claudin-1		
	Sense	AGGTCTGGCGACATTAGTGG
	Anti-sense	CGTGGTGTTGGGTAAGAGGT
MUC-2		
	Sense	AAAACTTTTGACGGCGATGT
	Anti-sense	TTCAGGTGCACAGCAAATTC
IL-17F		
	Sense	CCCATGGGATTACAACATCACTC
	Anti-sense	CACTGGGCCTCAGCGATC
IL-23 (α sub-unit)		
	Sense	CCTTCTCCGTTCCAAGATCCT
	Anti-sense	ACTAAGGGCTCAGTCAGAGTTGCT
